# Supplementary material for: A conformational benchmark for optical property prediction with solvent-aware graph neural networks
Source: Commun Chem. 2026 Feb 18;9:136. doi: 10.1038/s42004-026-01944-5 (PMC13031295; doi:10.1038/s42004-026-01944-5)
Supplement: Supplementary file 2 — Supplementary Information [file 42004_2026_1944_MOESM2_ESM.pdf]

Supplementary information: A Conformational Benchmark for Optical Property Prediction with Solvent-aware Graph Neural Networks

Denis Potapov[1,2*†], Sergei Rogovoi[1,3†], Kuzma Khrabrov[1], Konstantin Ushenin[1,4], Alexey Korovin[1], Anton Ber[1], Artur Kadurin[1,4,5,6], Artem Tsypin[1]

[1*]AIRI, Moscow, Russia.
[2]Moscow Institute of Physics and Technology, Moscow, Russia.
[3]Lomonosov Moscow State University, Moscow, Russia.
[4] Tomsk State University, Tomsk, Russia.
[5] Kuban State University, Krasnodar, Russia.
[6] ISP RAS Research Center for Trusted Artificial Intelligence, Moscow, Russia.

*Corresponding author(s). E-mail(s): potapov@airi.net;
Contributing authors: rogovoyserg@gmail.com; khrabrov@airi.net; ushenin@airi.net; korovin@airi.net; ber@airi.net; kadurin@airi.net; tsypin@airi.net;
†These authors contributed equally to this work.

Supplementary Methods

Pretraining setup

All 3D models were initialized from pretrained weights obtained on the the HOMO–LUMO gap prediction task. estimating the HOMO–LUMO energy gap. Hydrogen atoms were excluded from all molecular structures during both training and evaluation to reduce model complexity and computational cost.

PaiNN, DimeNet++, eSCN, and GemNet were pretrained by us on the full PCQM4Mv2 dataset, which contains DFT-calculated orbital properties for approximately 3.8 million molecules. UniMol+ was initialized from the official PCQM4Mv2 checkpoint released by the authors.

Evaluation protocol

To select optimal hyperparameters and model checkpoints, we used the validation set from the proposed ∇`Colors-3D` benchmark. The validation set was also used for early stopping and to adjust the learning rate (`ReduceLROnPlateau`). To ensure fair comparison, all models—including different architectures and conformer settings—were trained on identical data splits and corresponding molecular conformations.

Finetuning setup

The models were originally trained to predict HOMO-LUMO gap. To finetune on the ∇`Colors-3D`, we replace the regression head with a randomly initialized one. We then explore two strategies: 1) finetuning the model with a randomly initialized regression head end-to-end and 2) pretraining the regression head with a frozen backbone and then training the whole model end-to-end. We call these two setups “random” and “staged”.

While most models showed comparable performance under both protocols, the staged approach was crucial for UniMol+, enabling a more stable training process and improved final performance. This effect was consistent across all conformer types.

Training Hyperparameters

Supplementary Table 1: Key hyperparameters for the models used in this study. For the 3D models (PaiNN, DimeNet++, eSCN, GemNet), we report parameters for the `OneCycleLR` scheduler. An alternative `ReduceLROnPlateau` scheduler was also tested. For Chemprop, we report the hyperparameters from our best-performing configuration.

	**Chemprop**	**PaiNN**	**DimeNet++**	**eSCN**	**GemNet**	**UniMol+**
Training Hyperparameters						
Optimizer	Adam	AdamW	AdamW	AdamW	AdamW	Adam
Batch Size	128	32	64	32	32	6
Loss Function	MSE	MAE (L1)	MAE (L1)	MAE (L1)	MAE (L1)	UniMol+ loss
LR Scheduler	Cyclical	OneCycleLR	OneCycleLR	OneCycleLR	OneCycleLR	Polynomial Decay
Max LR	1.0×10^{-3}	5.0×10^{-4}	1.0×10^{-3}	1.0×10^{-3}	1.0×10^{-3}	8.0×10^{-5}
Epochs	300	450	450	450	450	—
Total Steps	—	—	—	—	—	300,000
Warmup	20 epochs	30% of steps	30% of steps	30% of steps	30% of steps	30,000 steps
EMA Decay	—	—	—	—	—	0.999
Model Architecture						
Layers	5 (depth)	3	6	8	4	6
Hidden size	482	128	128	128/256	256	768
Cutoff (Å)	—	5.0	5.0	8.0	12.0	—
Attention Heads	—	—	—	—	—	48

The main hyperparameters for training the models on absorption maximum wavelength prediction are detailed in Table 1. All models were trained with zero weight decay.

For the multi-target setup, we adopted the same hyperparameters but with key modifications to address the different prediction tasks. To handle the prediction of quantum yield (a value between 0 and 1), we applied a **logit transform** to the target values before calculating the loss. This transformation, defined as $\text{logit}(p) = \log(\frac{p}{1-p})$, maps the target to the range of real numbers, which is better suited for regression. To ensure numerical stability, the quantum yield values were clamped within the range $\left[3 \times 10^{-5}, 0.999 + 1 \times 10^{-5}\right]$ prior to transformation. In addition, we introduce loss weight coefficients. The coefficients were set to $[1, 1, 10]$ for the absorption wavelength, the emission wavelength, and the quantum yield, respectively, to align the quantum yield loss values with the absorption and emission loss values.

Chemprop implementation details.

All Chemprop models used in this study were trained with **Chemprop version 1.3.0**, obtained from the official Github release (https://github.com/chemprop/chemprop/tree/v1.3.0). This corresponds to the same Chemprop implementation used in the study *"Multi-fidelity prediction of molecular optical peaks with deep learning"* (DOI: 10.1039/D1SC05677H), ensuring consistency in molecular featurization and model behavior. Models trained with Chemprop v1.x are not directly compatible with Chemprop v2.x without running the official conversion script.

Supplementary Note 1: Additional experiments

Impact of Regression Head Initialization

We found that the effectiveness of head pretraining depends on the expressivity of the regression head.

Supplementary Table 2: Impact of regression-head initialization on *dft implicit-solvent* conformers. Columns show Train/Test MAE (nm) for **Random** vs. **Pretrained** heads

Model	Random		Staged	
	Train MAE ↓	Test MAE ↓	Train MAE ↓	Test MAE ↓
DimeNet++	3.5	25.1	2.8	18.5
ESCN	2.7	21.9	2.4	22.1
GemNet	1.8	20.6	1.8	21.3
Unimol+	5.4	22.0	5.5	18.4
UniProp	1.8	19.7	1.8	17.7

UniMol+, for example, utilizes a simple regression heads that is composed of two linear layers with GELU activation and layer normalization. UniMol+-based models consistently benefited from head pretraining, particularly when trained with lower-quality input geometries.

In contrast, GemNet's default regression head consists of an initial dense layer followed by multiple residual layers. Despite its expressive capacity, this configuration leads to overfitting when the backbone is frozen: the model achieves ∼5 nm MAE on the training set, but ∼30 nm on validation and test sets. This suggests that decoder expressiveness can harm generalization in transfer settings.

Less expressive regression heads used in models like DimeNet++, PaiNN, and eSCN demonstrated more stable behavior, but gained less from head pretraining. We hypothesize that this is due to their inability to leverage pretrained features.

Together, these findings highlight that the benefit of head pretraining depends on a balance between capacity and regularization. Pretrained heads are most helpful when the decoder is expressive enough to benefit from pretrained features, but not so complex as to overfit in low-data or frozen-backbone regimes.

Combined Cross-Validation Results

Supplementary Table 3: Consolidated cross-validation MAE metrics.

**Model**	**Target**	**Fold 1**	**Fold 2**	**Fold 3**	**Fold 4**	**Fold 5**	**Mean Val Loss**
**Single Target**							
UniProp	absorption	15.8 (*29.1*)	15.9	14.5	15.2	15.0	15.3 (*17.9*)
DimeNet++	absorption	19.1 (*32.5*)	21.0	17.2	17.8	17.7	18.6 (*21.3*)
Chemprop	absorption	26.0 (*41.8*)	22.1	19.1	20.5	21.0	21.7 (*24.9*)
**Multitarget**							
	absorption	15.3 (*29.8*)	14.3	14.9	16.3	17.2	15.6 (*18.5*)
UniProp	emission	20.3	17.9	20.3	19.7	21.3	19.9
	PLQY	0.17	0.16	0.16	0.15	0.15	0.16
	absorption	21.9 (*36.9*)	20.1	21.6	22.6	23.8	22.0 (*25.0*)
Chemprop	emission	29.6	26.0	28.7	27.6	29.9	28.4
	PLQY	0.18	0.18	0.18	0.16	0.18	0.17

This section reports full five-fold cross-validation metrics for the best 2D baseline, our proposed UniProp model and the second best performing model – DimeNet++ with solvent embedding. As DimeNet++ performed worse than UniProp in single target setting, we did not train in multitarget setting. We first train both models in a single-task setting to predict only the peak absorption wavelength. We then train them in a multitask setting in which, for each chromophore–solvent pair, the models jointly predict the peak absorption wavelength, the peak emission wavelength, and the photoluminescence quantum yield (PLQY). For **Fold 1**, we report two MAE values due to the betaine dye36 issue described in Section 3.3. The italicized MAE is computed on the full fold. The non-italicized MAE is computed on the same fold after excluding the ∼ 200 samples in which the chromophore is betaine dye36.

Conformer calculation times

In this section, we provide details on time it takes to calculate optimized geometries using different levels of quantum theory.

Supplementary Table 4: CPU times for conformer calculations using different methods. Times are in seconds, minutes and hours.

Method	Mean	Median	Max	Total time
Orca Solvent	1493.98 s (24.90 min)	373.20 s (6.22 min)	6554.51 min	10940.93 h
Orca Vacuum	1127.32 s (18.79 min)	342.13 s (5.70 min	1939.08 min	4301.04 h
XTB Vacuum	27.54 s (0.46 min)	9.01 s (0.15 min)	25.32 min	118.65 h

Training curves

In this section, we provide full training plot for the solvent-aware variants of 3D GNNs. Each row corresponds to a single model. The left subplot in each row depicts training loss, whereas the right subplot depicts the validation loss. In each subplot, we provide three curves, corresponding to different conformation types.

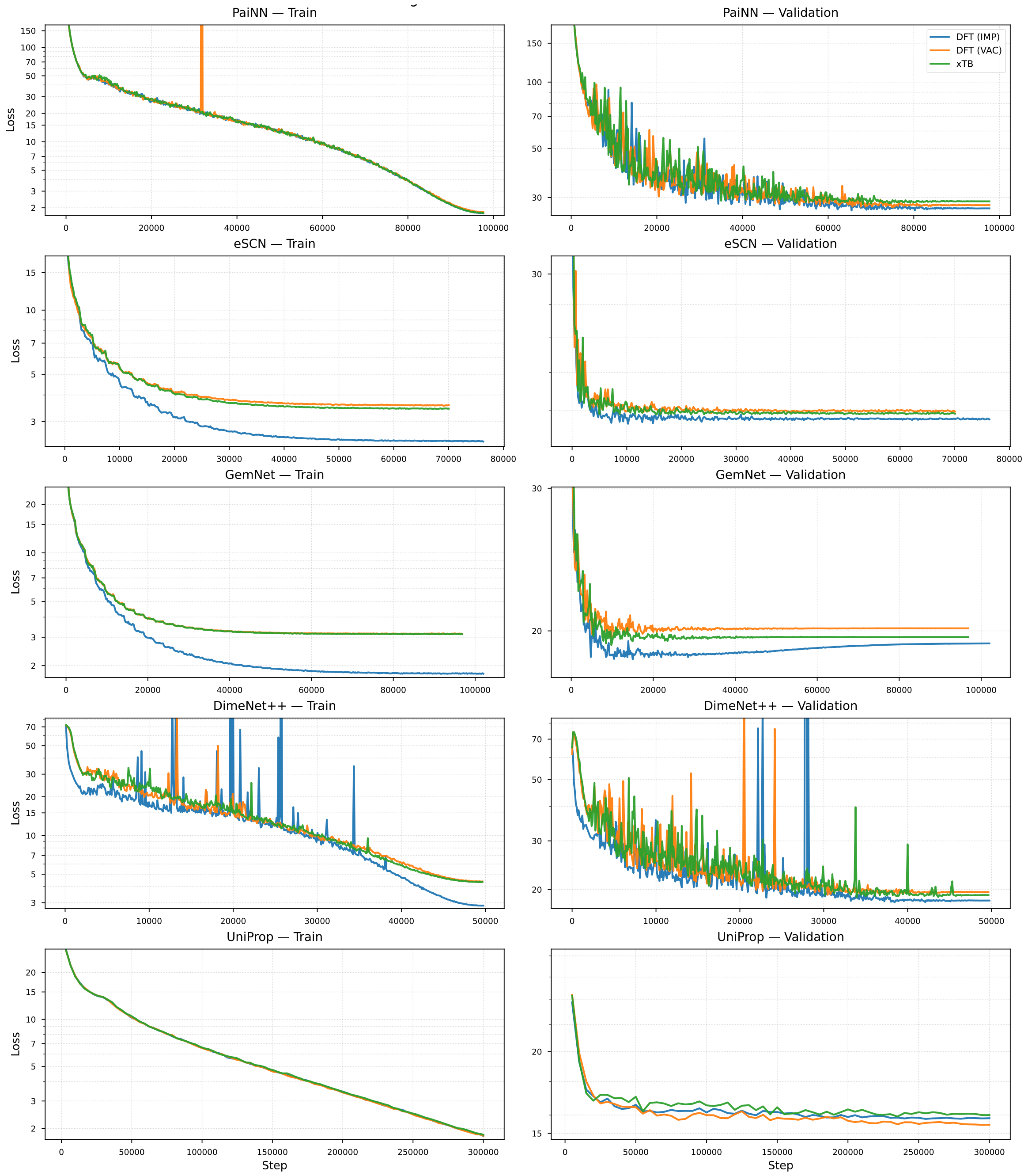

Supplementary Figure 1: Training curves for the solvent-aware variants of 3D GNNs. The y-axis is log-scaled.

Supplementary Note 2: Uniprop error analysis

To address whether particular classes of chromophores benefit most from including 3D information, and to understand which molecules remain difficult despite having 3D conformations, we performed a

Supplementary Table 5: Pearson correlation of molecular descriptors with UniProp error and improvement over ChemProp.

Descriptor	Error (nm) r	Error (freq) r	Improv. (nm) r	Improv. (freq) r
MaxTanimoto	-0.208	-0.216	-0.164	-0.223
MolWt	0.123	0.004	-0.021	-0.106
RotatableBonds	0.058	-0.029	0.013	-0.063
NumRings	0.086	-0.000	-0.070	-0.127
FractionCSP3	0.053	-0.002	0.116	0.071

post hoc analysis on the held-out test set. We quantify (i) the per-molecule absolute prediction error of UniProp and (ii) the improvement over the 2D Chemprop baseline, and relate these quantities to a compact set of molecular descriptors and dataset coverage proxies.

Throughout this section, we analyze errors both in the native wavelength representation (nm) and in the corresponding frequency domain. The frequency-domain representation is obtained by converting wavelengths λ to optical frequency (or equivalently, energy) via $\nu = c/\lambda$, where c is the speed of light. While wavelength errors are commonly reported in experimental spectroscopy, expressing errors in frequency units reduces nonlinear distortions inherent to the inverse relationship between wavelength and energy, and enables a more physically uniform comparison across the spectral range.

Correlation with molecular descriptors and training set similarity

Table 5 summarizes Pearson correlations between molecular descriptors and both UniProp error and the improvement over Chemprop. Overall, these correlations are weak, suggesting that residual errors are not strongly explained by simple size or flexibility descriptors within the benchmark. The strongest trend is a moderate negative correlation with maximum Tanimoto similarity to the training set, consistent with an out-of-distribution (OOD) effect: molecules less similar to the training distribution tend to have larger errors and smaller improvements.

We additionally note a weak positive correlation between molecular weight and the absolute error measured in wavelength units (nm), while this correlation essentially vanishes in the frequency domain. This behavior is consistent with the known physical trend that larger conjugated chromophores tend to absorb at longer wavelengths (smaller electronic gaps), which can induce a mild size dependence in absolute errors when measured in nm. Expressing the target in frequency (or energy) units mitigates this unit-dependent effect and more directly reflects model performance across chromophore sizes.

Most challenging molecules and molecules benefiting most from 3D information

To provide qualitative insight into where the model succeeds or fails, we visualize the ten molecules with the largest UniProp errors (Fig. 3) and the ten molecules with the largest improvement of UniProp over Chemprop (Fig. 4). These examples illustrate that both high-error and high-improvement cases span diverse chromophore scaffolds, and do not show a clear association with any specific chromophore family, molecular size, or degree of flexibility, in agreement with the weak correlations observed in Table 5.

Do the largest errors require conformer ensembles?

To test whether the largest remaining errors could be explained by insufficient conformational sampling, we performed CREST conformer searches with GFN-FF for the ten molecules with the largest UniProp errors. We evaluated UniProp predictions using the lowest-energy conformer, a Boltzmann-weighted conformer ensemble, and a simple mean ensemble, and compared these to the original single-conformer evaluation. Table 6 reports the mean absolute error across these ten molecules for each protocol; we do not observe a systematic improvement over the original conformer choice, suggesting that for these cases the dominant error source is unlikely to be a suboptimal local minimum found during conformer generation.

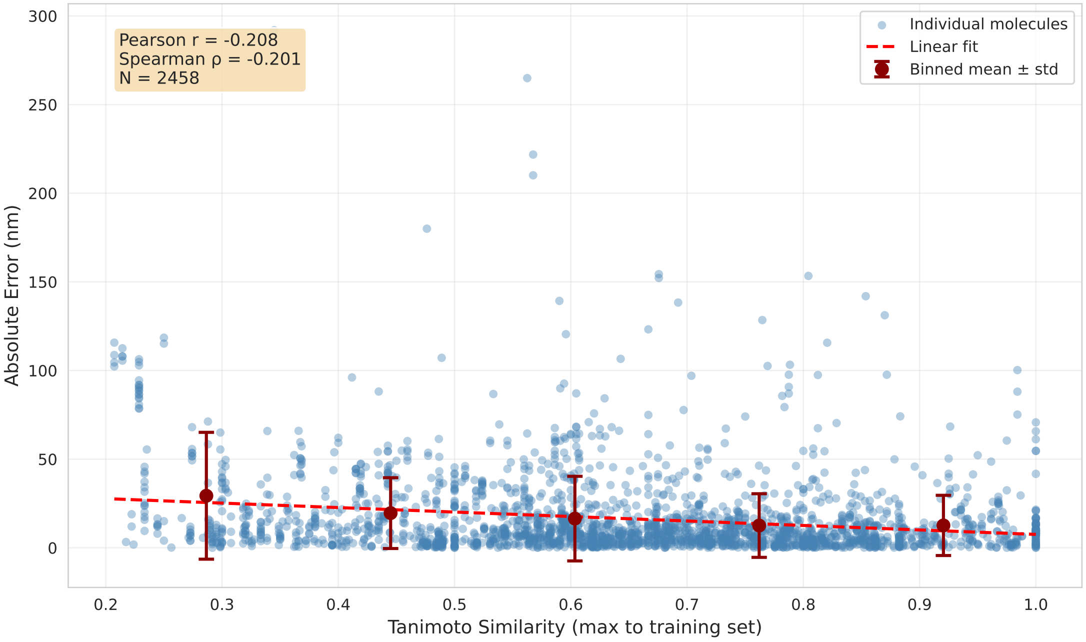

Supplementary Figure 2: UniProp absolute error (nm) on the test set versus maximum Tanimoto similarity to the training set (coverage/OOD proxy). Each point corresponds to one molecule. The weak but consistent negative trend (Pearson and Spearman correlations shown in the plot) indicates that molecules farther from the training distribution tend to be predicted less accurately.

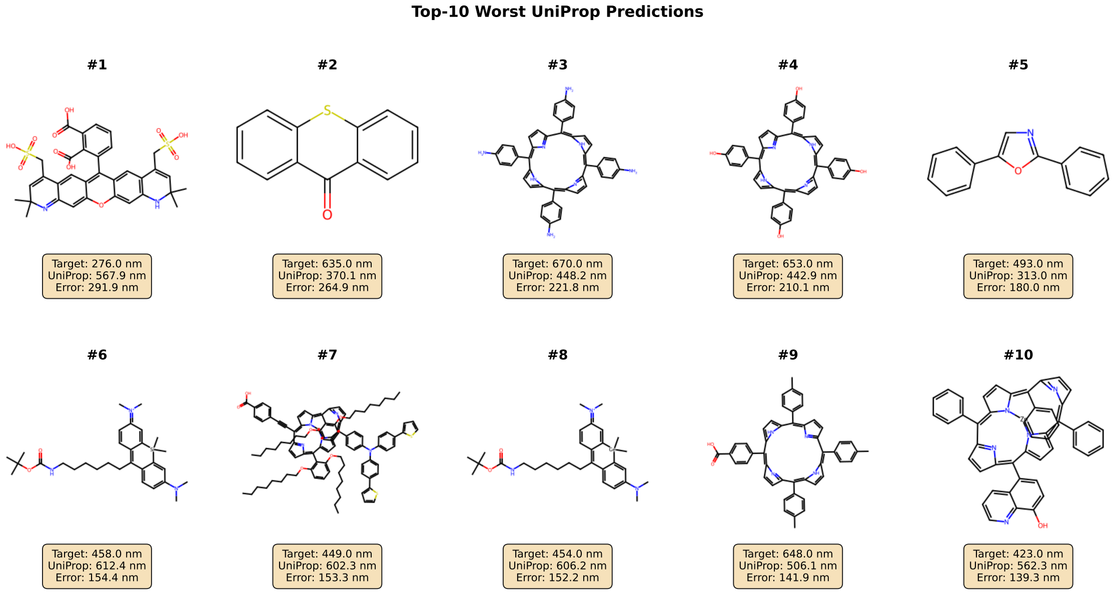

Supplementary Figure 3: Top-10 molecules with the largest UniProp absolute error on the test set (nm). For each molecule we show the 2D structure and report target wavelength, UniProp prediction, and absolute error.

Supplementary Table 6: Summary of mean error (nm) for CREST conformer ensemble methods on 10 molecules.

	Original	Best Conformer	Boltzmann Ensemble	Mean Ensemble
Mean Error (nm)	173.64	176.92	176.67	175.80

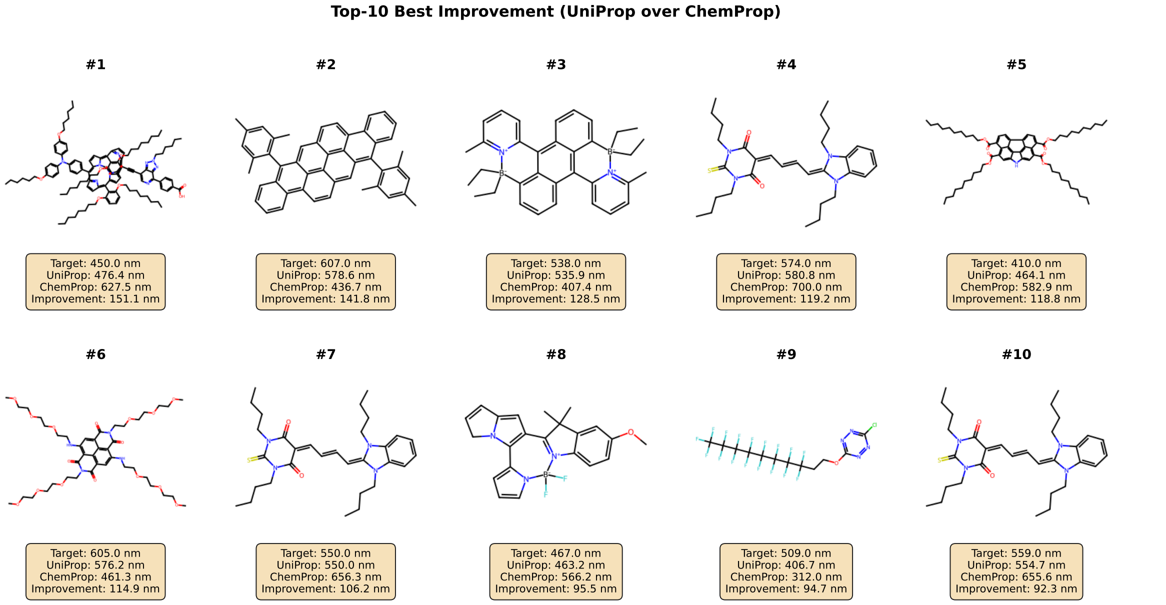

Supplementary Figure 4: Top-10 molecules with the largest improvement of UniProp over Chemprop on the test set (nm). For each molecule we show the 2D structure and report target wavelength, UniProp prediction, Chemprop prediction, and the improvement in absolute error (Chemprop error minus UniProp error).
